# Evaluation of Patient Satisfaction Using the EORTC IN-PATSAT32 Questionnaire and Surgical Outcome in Single-Port Surgery for Benign Adnexal Disease: Observational Comparison with Traditional Laparoscopy

**DOI:** 10.1155/2013/578392

**Published:** 2013-11-25

**Authors:** Alessandro Buda, Marco Cuzzocrea, Luca Montanelli, Paolo Passoni, Lorena Bargossi, Romina Baldo, Luca Locatelli, Rodolfo Milani

**Affiliations:** Department of Obstetrics and Gynecology, University of Milano-Bicocca, San Gerardo Hospital, Via Pergolesi 33, 20900 Monza, Italy

## Abstract

Laparoscopic surgery has been demonstrated as a valid approach in almost all gynaecologic procedures including malignant diseases. Benefits of the minimally invasive approach over traditional open surgery have been well demonstrated in terms of minimal perioperative morbidity and reduced postoperative pain and hospital stay duration, with consequent quick postoperative recovery (Medeiros et al. (2009)). Single-port surgery resurfaced in gynaecology surgery in recent years and renewed interest among other surgeons and within the industry to develop this field (Podolsky et al. (2009)). Patient satisfaction is emerging as an increasingly important measure of quality which represents a complex entity that is dependent on patient demographics, comorbidities, disease, and, to a large extent, patient expectations (Tomlinson and Ko (2006)). It can be broadly thought to refer to all relevant experiences and processes associated with health care delivery (Jackson et al. (2001)). In this study we aim to compare single-port surgery (SPS) with conventional laparoscopy in terms of patient satisfaction using the EORTC IN-PATSAT32 questionnaire. We also evaluate the main surgical outcomes of both minimally invasive approaches.

## 1. Introduction

Laparoscopic surgery has been demonstrated as a valid approach in almost all gynecologic procedures including malignant diseases. Benefits of the minimally invasive approach over traditional open surgery have been well demonstrated in terms of minimal perioperative morbidity and reduced postoperative pain and hospital stay duration, with consequent quick postoperative recovery [1].

Single-port surgery resurfaced in gynecology surgery in recent years, since in 2007 Podolsky and colleagues reported their experience with single-port cholecystectomy and renewed interest among other surgeons and within the industry to develop this field [2].

Laparoendoscopic single-site surgery represents another innovation in minimally invasive gynecologic surgery whose goal could be to further improve not only the surgical or physical outcomes but also, and more generally, the quality of health care.

Patient satisfaction is emerging as an increasingly important measure of quality [3] that seems to be independent from other clinical and surgical outcomes.

Patient satisfaction can be defined as the extent to which an individual's experience in health care matches his or her expectations. Obviously, patient satisfaction is a very complex entity that is dependent on patient demographics, comorbidities, disease, and, to a large extent, patient expectations. It can be broadly thought to refer to all relevant experiences and processes associated with health care delivery [4].

In this study we aim to compare single-port surgery (SPS) with conventional laparoscopy in terms of patient satisfaction using the EORTC IN-PATSAT32 questionnaire. We also evaluate the main surgical outcomes of both minimally invasive approaches.

## 2. Materials and Methods

This single-institution observational study included women with benign adnexal disease who underwent laparoscopic minimally invasive surgery in the Gynecology Department at San Gerardo Hospital, University of Milano-Bicocca, Monza, between November 2011 and December 2012. Clinical inclusion criteria were cystic adnexal masses with benign clinical features and major diameters equal to or less than 10 cm, prophylactic adnexal remove in patients with BRCA mutations, and ectopic pregnancies.

We evaluated two groups of women who underwent single-port laparoscopic surgery (SPS) or traditional laparoscopic surgery carried out by the same surgeon. This observational study was not conceived as a clinical trial/intervention study, and, therefore, women were not assigned into study and control groups by the investigators. The study was conducted in accordance with the Declaration of Helsinki and an Institutional Review Board approval was obtained. All women provided a written informed consent.

### 2.1. Data Collection

Demographic and clinical characteristics were recorded for all patients (see details in Table 1). All surgical outcome data such as operative time, estimated blood loss (EBL), conversion rate to standard laparoscopy or laparotomy, duration of immobilization, type and duration of postoperative analgesia, and the EORTC IN-PATSAT32 satisfaction questionnaire were registered in a dedicated electronic data base. All the included surgical procedured were performed by a single team with the same senior surgeon (AB). Long-term complications and histological findings were also entered in the electronic database.

### 2.2. Surgical Procedure in Single-Port Cases

The SILS port device (SILS, Covidien, Mansfield, MA) and the TriPort single-port device (laparoendoscopic single-site surgery, Olympus Winter & IBE GMBH, Hamburg, Germany) were used in all cases of single-site surgery. Articulating instruments were used in every SPS performed. In order to access the feasibility of single-port surgery, a standard 5 mm umbilical diagnostic laparoscopy was performed before the insertion of the single-port device in the study group. Once pneumoperitoneum was achieved (12 mmHg), intra-abdominal visualization was obtained with a 5 mm, 50 cm long 30° telescope. Working straight 5 mm instruments were inserted into the remaining two ports of the single-port devices.

Specimen removal was achieved within an endobag inserted in the 12 mm port of the trocar. Each layer of the umbilical access port was separately sutured; in particular the abdominal fascia was closed by singular separate suture. Skin was repaired with rapid absorbable suture.

Postoperatively, both groups of patients were administered identical analgesic treatment. All women received Ketoprofen 100 mg in a short intravenous infusion, twice/daily, plus Paracetamol 1000 mg intravenously three/times daily. After 24 hours (postoperative day 1) analgesic drugs were administered on demand and the daily dose did not exceed 200 mg of Ketoprofen and 3000 mg of Paracetamol.

### 2.3. EORTC IN-PATSAT32 Questionnaire

The EORTC IN-PATSAT32 is composed of 32 items assessing patient perception of the quality of hospital doctors and nurses, as well as selected aspects of the care organization and hospital environments that are relevant. This questionnaire was developed according to the guidelines and procedures recommended by the EORTC QL Group [5]. Items are all rated on a five-level Likert scale with the category labels “poor,” “fair,” “good,” “very good,” and “excellent.” Scores for all domains and single-item measures ranged from 0 to 100.

Before their discharge from the hospital, women were asked to complete the satisfaction questionnaire EORTC IN-PATSAT32. All patients were informed of the objectives and procedures of the study. After patient's acceptance of participation, questionnaires were distributed in the Gynecology Department, to be completed at home within six weeks after discharge. A reminder letter was sent if the questionnaire was not returned and, where necessary, was followed by a telephone reminder.

### 2.4. Statistical Analysis

Continuous variables are reported as mean and standard deviation, while discrete variables are reported as percentages of the total. Differences between the two groups were analyzed using a *t*-test. Percentages were compared using Fisher's exact test. Probability values were considered statistically significant with *P* < 0.05. The Shapiro-Wilk test has been used to verify normality of the study population.

## 3. Results

A total of 72 women were enrolled in the study. The SPS group included 37 patients, whereas 35 patients were in the standard control group. Baseline characteristics are described in Table 1. There were no significant differences between the groups in terms of age, BMI, previous pelvic surgery and type of surgery, estimated blood loss, conversion rate, and postoperative fever. There were no intraoperative complications. In one woman of the SPS group (2.7%) with an endometriotic cyst, because of a large bowel tenacious adherence to the uterus, one 5 mm additional trocar in the iliac fossa was required to complete the planned surgery.

Analysis of the questionnaire scores did not reach any statistically significant difference between the two groups (75 for SPS versus 78 for the control group; *P* = 0.14) and was independent of the physician and surgical outcomes evaluated between groups. This result was maintained when data were analyzed in the doctors' and nurses' subgroups of the questionnaire. Median satisfaction scores were good in both groups, with a general satisfaction score item “very good” or “excellent” in 75% of both groups. Satisfaction scores missing data were only 3% for both groups. The lowest satisfaction score was reported for “ease of access” in both groups (see details in Table 3). The observed standard deviations were not fairly large, ranging from 7.23 (nurses' items) to 10.75 (doctors' technical skills items) for standard laparoscopy and from 7.51 (nurses' items) to 14.03 (doctors' technical skills items) for SPS. Moreover, scores of the IN-PATSAT32 scales demonstrated very close to normal distribution with Shapiro-Wilk estimates ranging from 0.80 to 0.95.

Mean duration of surgery in the SPS group was significantly lower than that of the surgery performed by traditional laparoscopy (67 versus 93 minutes; *P* = 0.002). Moreover, mean operative time of SPS was significantly lower in the final 19 patients, compared to the first 18 cases (50 versus 68 minutes; *P* = 0.04).

Median postoperative analgesic administration was significantly lower in the SPS group (18 versus 24 hours; *P* = 0.0002). No complications occurred in all patients in the follow-up period, except one case of serosity from the umbilical wound without infection in a girl who had undergone SPS with adnexal cystectomy for a left dermoid cyst; no hospitalization was required and the serosity resolved within few days with medication and no further complications occurred. Surgical outcomes are shown in Table 2.

## 4. Discussion

Single incision laparoscopic surgery represents a rapidly evolving minimally invasive approach that recently has been widely adopted in gynecology.

Even if promoted as a new technique, it has been adopted for several years in gynecologic surgery, since a single-port laparoscopic tubal ligation was performed by Dr. Wheeless Jr. in 1972 [6]. 

In the last couple of years, a large amount of prospective reports have demonstrated the feasibility of single-port surgery for both benign and oncologic conditions, with either traditional or robotically assisted laparoscopy [7–10].

The disadvantages of single-port surgery are mainly the added cost and the technical challenges due to limitation of working space. However loss of triangulation and instrument clashing have been progressively minimized by some practical adjustments, such as use of dedicated, articulated, and longer instruments that have the advantage to avoid crossing outside the abdominal cavity.

The most apparent benefit of single-port laparoscopy is the improved cosmetic result particularly in young patients, because it only leaves a hidden scar in the umbilicus. However possible advantages of single-site surgery can be extended to reduce operative complications related to trocar insertion, easier surgical specimen removal through a larger incision, and reduction of postoperative pain. As already demonstrated in studies with traditional laparoscopy reducing the size of the ancillary ports seems to reduce immediate postoperative pain [11]. Considering SPS, it could be assumed that having no ancillary ports would likewise decrease postoperative pain.

Nevertheless, a recent systematic review and meta-analysis published by Murji et al. [12] did not find any difference in the risk of complications between single-port surgery and conventional laparoscopy approaches in gynecologic surgery. Included studies suggest that SPS may have longer operative time for adnexal surgery but not for hysterectomy. Because of paucity of data and lack of uniform reporting, the meta-analysis did not find significant effects on other surgical outcomes such as postoperative pain, change in hemoglobin, length of hospital stay, and scar cosmesis.

Evaluation of the impact of surgery on reduction of postoperative pain is always hard, particularly in case of noninvasive surgery with a less extensive procedure that usually demonstrates a quick postoperative recovery. Moreover each patient's interpretation of the VAS score is highly individualistic given difference in pain tolerance and perception. A recent article of Galloway et al. [13] found that the impact of postoperative mean VAS scores was extremely limited, predicting neither pain medication utilization nor frequency of pain assessment in a gynecologic surgery group of patients.

Surgeon satisfaction is also a key element that needs to be evaluated. The exact number of procedures required to complete the learning curve in regard to operative time depends on the surgeon's skills and ability matured with traditional laparoscopy and his adaptability to the single-port procedure to quickly and easily overcome some technical challenges. A learning curve as low as 5 cases has been reported in general surgery with single-port cholecystectomies [14].

In our experience we found a significantly shorter operative time for the final 19 patients who underwent SPS compared to that of the first 18 patients (50 versus 68; *P* = 0.04), which confirms the presence of a learning curve as already demonstrated in the available literature [15, 16].

This study started after a training experience of a dedicated surgical staff with single-port surgery. During the learning curve period we tried to use as long as possible the standard laparoscopic instruments; thereafter we introduced some articulating instruments such as graspers, scissors, and dissector (Roticulator Covien, Mansfield, MA) which facilitated execution of surgical procedures, primarily avoiding instruments clashing outside the patients.

Interpretation of this results can be also related to a sort of selection of patients in the SPS group. However, the careful case selection and a low threshold of conversion to conventional laparoscopic surgery are essential in this type of surgery, which is also recommended by the LESSCAR (the Laparoendoscopic Single-Site Surgery Consortium for Assessment and Research) multidisciplinary working group that coordinates and promotes researches in the field of single-port surgery [17].

Our study has some limitations and from our results we cannot draw firm conclusions. Moreover, even if the analysis of the surgical outcomes found a statistical significant difference in favor of single-port surgery in terms of duration of immobilization, early discharge of patients, and duration of postoperative analgesia, we must take into account that patients in the SPS group have been carefully selected for this type of surgery. Single-port surgery seems to improve cosmetic results and is quite appreciated and positively accepted by young patients. In our opinion, evaluating patient satisfaction seems to be equally important as analyzing mainly postoperative variables.

Usually surgeons have a kind of “natural bias” that patient's satisfaction is directly related to a surgical or physical outcome. Although surgeon's satisfaction often coincides with the patient's satisfaction, the projection of our satisfaction in the patient may not necessarily be always correct.

The satisfaction scores from the EORTC QLQ-PATSAT32 were used with our intent to address patient satisfaction with caregiver staff and overall satisfaction. Our observation is similar to the results in the article of Avery et al. performed in a subgroup of oncologic patients [18]; indeed their surprising finding was that patient satisfaction was independent of quality of life scores or surgical outcome.

Even if surgical and physical outcomes were almost excellent in all cases of SPS and control patients, we did not find any significant difference between the two groups in terms of satisfaction scores. The lowest satisfaction score was reported for “the ease of access” in both groups. Moreover, regarding the satisfaction with doctors, the lowest score was reported for “their willingness to listen to all of your concerns” in the control group and for “the time they devoted to you during visits/consultations” in the SPS group. Looking at the satisfaction with nurses, the lowest score was for “the information they gave you about your medical tests” and for “The information they gave you about your care,” thus highlighting areas for improvement that may be critical to good team working and in prospective, by modifying the staff behavior, patient satisfaction may probably improve.

Our observations have been already demonstrated in subgroups of oncology patients, confirming that improving communication between health care employers is important and essential to ensure high quality of care [19, 20].

Notwithstanding, even as we know that the most important limit of our study is the limited sample size and the nonrandomized study design to state firm conclusions, we can suppose that SPS is not inferior to traditional laparoscopy in performing adnexal surgery also in terms of patient satisfaction.

In our experience, results obtained in this study with the somministration of the EORTC IN-PATSAT32 represent a tool that must be further verified prospectively in future large series of cases together with the surgical outcomes to help our planning to further improve health services and care.

Finally, the approach to evaluating outcomes of treatment with patient-based measures provides useful feedback to improve both patient satisfaction and caregiver coordination and teamwork. This goal will take time, but probably it will be no wasted time.

Future prospective studies are needed to address this important issue by investigating the responsiveness of the satisfaction questionnaire to modify the structure and process of the health care system and also to establish and maintain a healthy doctor-patient relationship.

## Figures and Tables

**Table 1:** Patients characteristics.

	Single-port surgery (SPS) (*n* = 37)	Standard laparoscopy (*n* = 35)	*P*
Median age, yes (range)	42 (16–79)	33 (12–69)	0.36
Median BMI, median (range)	23 (17.2–34.9)	20.5 (17.8–35.3)	0.53
Surgical indications, no (%)			
Cystectomy	24 (65)	26 (74)	n.s
Salpingo-oophorectomy (including BRCA mutation)	12 (32)	6 (17)	0.65
Tubal pregnancy	1 (3)	3 (9)	n.s
Parity, no (range)	0.84 (0–5)	0.37 (0–3)	<0.001
Previous abdominal and/or pelvic surgery, no (%)	17 (46)	14 (40)	0.52
Greatest cyst diameter, median, mm (range)	63 (0*–90)	67 (0*–105)	n.s

*Prophylactic salpingo-oophorectomy.

**Table 2:** Surgical outcomes.

	Single-port surgery (*n* = 37)	Standard laparoscopy (*n* = 35)	*P*
Operative time, median (range), min	67 (45–130)	93 (42–163)	0.002
EBL, median (range), mL	10 (0–100)	22 (0–150)	N.S
Conversion to LPS or LPT, no	0	0	—
Intraoperative complications, no	0	0	—
Postoperative complications, no	1 (3.7%)	0	—
Hospital stay, median (range), days	1 (1–3)	2 (1–4)	0.002
Duration of immobilization, median (range), hours	10 (4–24)	13 (4–48)	0.003
Postoperative analgesia, median (range), hours	18 (7–36)	24 (6–56)	0.0002
Salpingo-oophorectomy (mono- or bilateral), no (%)	12 (32%)	6 (17%)	0.65

EBL: estimated blood loss; LPS: laparoscopy; LPT: laparotomy.

**Table 3:** Patient satisfaction score results (*n* = 72; completed questionnaire *n* = 70; missing data 3%).

	Single-port surgery (*N* = 37)	Standard laparoscopy (*N* = 35)	*P*
Satisfaction score 1–32, median (range)	75 (62–99)	78 (66–98)	0.14
Satisfaction score 1–11, median (range)	78 (70–99)	82 (77–98)	0.89
Satisfaction score 12–22, median (range)	75 (65–78)	78 (72–84)	0.41
Satisfaction score 23–32, median (range)	71 (62–87)	72 (66–82)	0.16
